# Significance of Diagnostic Findings of Placenta Percreta by Magnetic Resonance Imaging and Pathological Correlation

**DOI:** 10.7759/cureus.75685

**Published:** 2024-12-13

**Authors:** Francisco I Jara-García, Oscar A Regalado-Morales, Ángeles E González-Recio, Adrián A Negreros-Osuna

**Affiliations:** 1 Radiology Department, Hospital Regional Instituto de Seguridad y Servicios Sociales de los Trabajadores del Estado Monterrey, Universidad de Monterrey, Monterrey, MEX

**Keywords:** diagnosis, histopathology, magnetic resonance imaging (mri), placenta accreta spectrum (pas), placental accreta, placenta percreta

## Abstract

Placenta percreta is a rare form of disorder found in the spectrum of placenta accreta and represents a considerable cause of maternal complications with an increase in mortality. The radiologist's role is essential due to the support of images acquired by magnetic resonance imaging, given their high sensitivity and specificity to predict the degree of placental invasion in substitution or accompaniment of the ultrasound study between 28 and 32 weeks of gestation. We present the case of a 29-year-old patient who was in her third pregnancy with a history of two cesarean sections at the ISSSTE Regional Hospital in Monterrey, Nuevo León.

## Introduction

Obstetric hemorrhage is one of the main causes of maternal morbidity and mortality in Mexico, and the severe postpartum form represents approximately 10% with a mortality of 1:1000 cases in developing countries [1]. An important risk factor for obstetric hemorrhage is the placenta accreta spectrum (PAS), which is defined as the pathological or abnormal implantation of the placenta in the myometrium with the hypothesis raised by a defect in the endometrium-myometrium interface, which causes an alteration in normal decidualization resulting in trophoblastic migration, abnormal adherence, and invasion of placental chorionic villi in the adjacent myometrium. There are multiple risk factors for placental accreta disorder, such as multiparity, history of cesarean sections, placenta previa, endometrial ablation, advanced maternal age, infertility, and assisted reproduction techniques [2]. 

The incidence of placenta accreta spectrum has increased over time in the last 50 years given the high number of births via cesarean section, reaching three cases per 1000 pregnancies worldwide and one in 650 in our country [3].

Imaging studies in placenta accreta disorder with MRI are indicated when the ultrasound evaluation is equivocal or in cases where ultrasound has already made a definitive diagnosis for planning cesarean section and peripartum hysterectomy. Because of the high risk involved, the radiologist must be familiar with the recommended protocols for performing magnetic resonance imaging and the main examination findings for appropriate management. Recent meta-analyses demonstrate an overall sensitivity of MRI between 86.5% and 94.4% with a specificity of 96% to 98.8% in predicting the depth of placental invasion. We must interpret these data with caution due to pretest bias, as only those with suspected placenta accreta disorder undergo an MRI study. Skill and experience in image interpretation may not be available in all hospital settings; however, a 2017-2018 international survey of FIGO expert panel members showed increasing use of MRI in the diagnosis of PAS disorders, with both ultrasound and MRI being used up to 61% [2,4].

## Case presentation

This is a 29-year-old patient with three gestations, two cesarean sections (in 2018 due to death of unknown cause and in 2020 due to a previous cesarean section), no abortions, last menstruation date October 26th, 2023, and expected day of delivery on August 1, 2024. She did not report any significant personal pathological history and attended seven prenatal consultations, the first one being at six weeks of gestation. An obstetric ultrasound was performed on April 16th, 2024, reporting a product in cephalic, longitudinal presentation and a central placenta previa with grade 1 accreta phenomenon, umbilical cord with two arteries and one vein.

Magnetic resonance imaging findings

On June 10th, 2024, the pelvic magnetic resonance imaging study was performed with Siemens Healthineers MAGNETOM Amira 1.5T equipment using T2, T2FS, STIR, and DWI sequences in sagittal, axial, and coronal planes, respectively.

**Figure 1:**
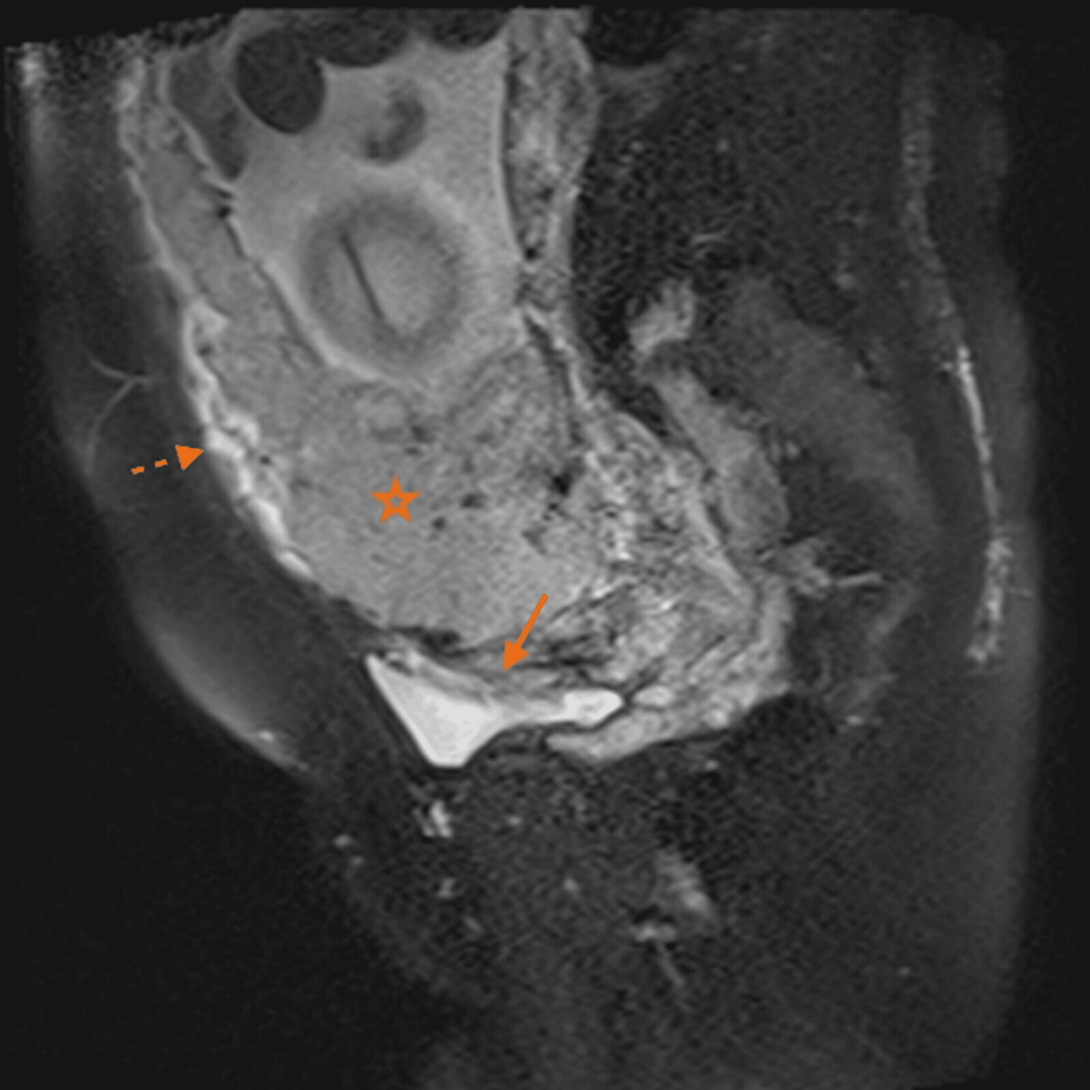
Magnetic resonance imaging in T2 sequence with fat saturation in the sagittal plane. Placenta (star) with irregular morphology, heterogeneous due to the presence of hypointense bands and signal voids. The lower uterine segment is widened. Loss of continuity of the retroplacental hypointense line due to placental invasion (arrow) into the bladder. The myometrium is observed preserved in the anterior uterine segment (dashed arrow).

**Figure 2:**
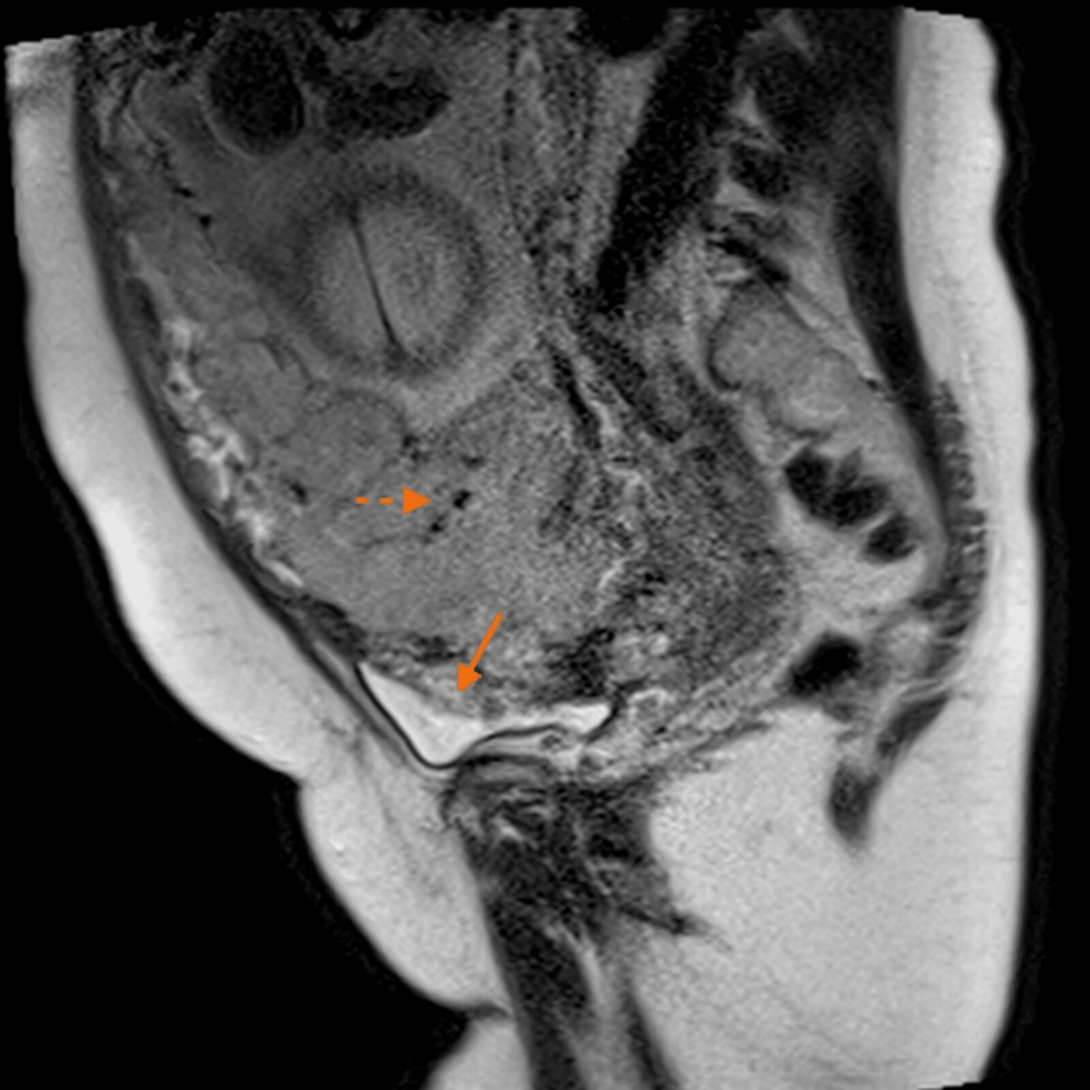
Magnetic resonance imaging in the sagittal plane in T2 sequence without fat saturation. We observe the loss of the hypointense bladder wall secondary to placental invasion (arrow). Flow voids (dashed arrow) are observed in relation to anomalous intraplacental vessels.

**Figure 3:**
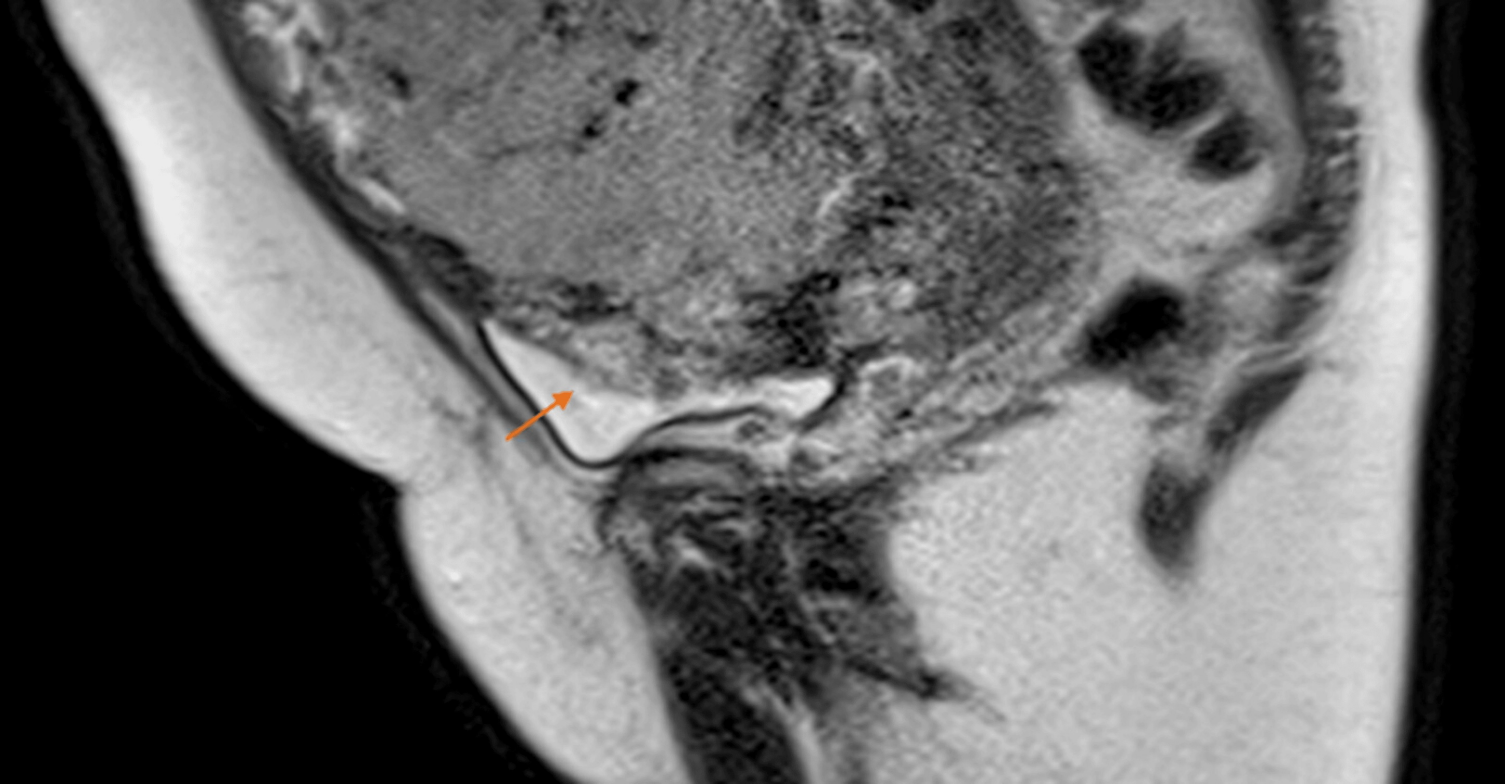
Magnetic resonance imaging in the sagittal plane in the T2 sequence without fat saturation extended towards the bladder. The loss of the hypointense line of the anterior bladder wall is observed secondary to the presence of a focal exophytic mass highly suggestive of placenta percreta.

**Figure 4:**
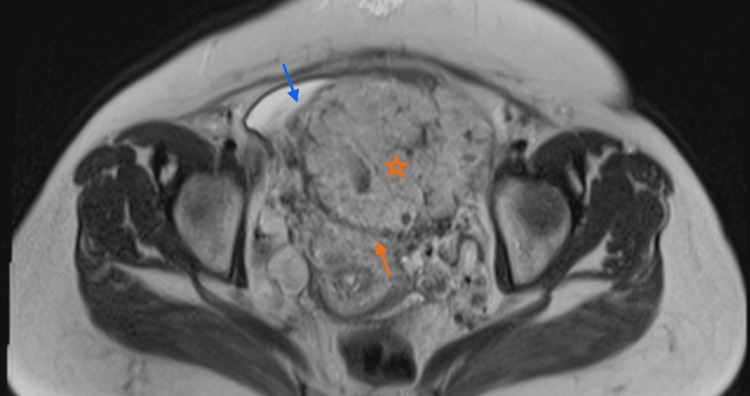
Magnetic resonance imaging in T2 sequence without fat saturation in the axial plane. Placenta with a heterogeneous appearance (star) with a focal exophytic mass at the level of the uterovesical junction, extending towards the bladder (blue arrow). In the posterior segment, the preserved hypointense retroplacental line is observed (orange arrow).

**Figure 5:**
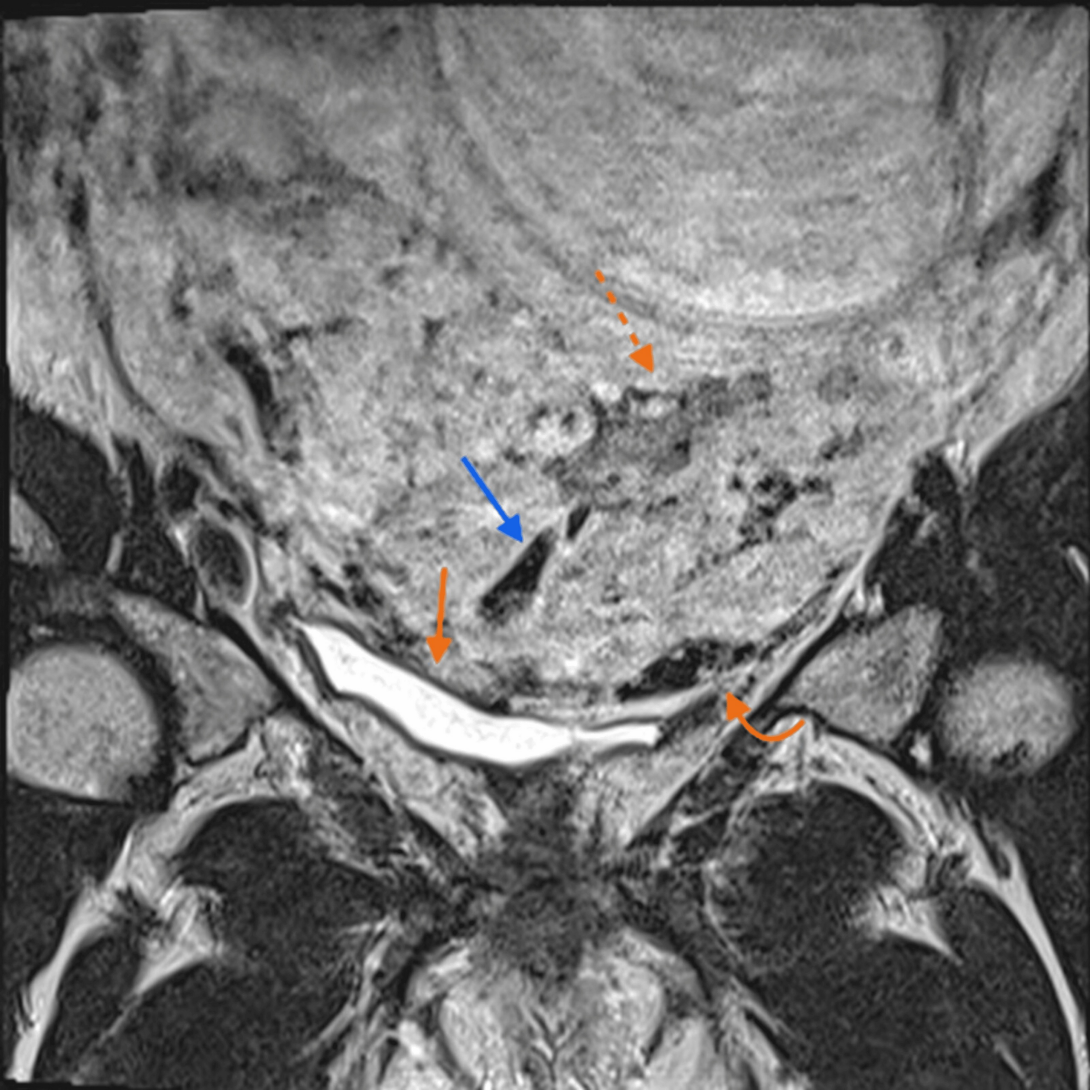
T2-weighted magnetic resonance image without fat saturation in the coronal plane. Placenta with a heterogeneous appearance due to the presence of hypointense bands (dotted orange arrow) and flow voids related to anomalous intraplacental vessels (blue arrow) as well as anomalous vessels of the placental bed (curved orange arrow). At this level, the bladder wall is intact, preserving the normal hypointense line of the bladder wall (orange arrow).

The uterus presented a singleton pregnancy with a left occiput presentation. Significant myometrial thinning was observed with loss of the hypointense retroplacental line in the lower right uterine segment (Figures 1, 2), which was accompanied by a focal exophytic mass and invasion of the placental tissue into the myometrium, breaking the serous margin with extension towards the bladder wall with loss of its normal hypointensity (Figures 3, 4). The placenta had irregular morphology and heterogeneous signal intensity with multiple intraplacental hypointense bands and some enlarged and tortuous intraplacental vessels (Figure 5). There was no free fluid in the abdomen or pelvic cavity. The adnexa were of normal characteristics.

Pathology findings

During the surgery, placental infiltration with invasion of the myometrium and uterine serosa was observed. A corporal fundal hysterotomy was performed, liquor was clear and adequate, and the fetus was extracted by podalic extraction. Subsequently, a hysterectomy was performed, observing the bladder adherent to the anterior wall of the uterus with placental infiltration. There was difficult-to-control bleeding in the bladder vessels, which required double clamping of the uterine vessels with subsequent hemostasis of the bladder dome and bilateral salpingectomy, quantifying a total bleeding of 2500 cc.

Macroscopic description

Uterus weighing 1,200 grams and measuring 17 x 13 x 11 cm, partially covered by light brown serosa, anterior surface with suture threads and soft consistency, exocervix measuring 4 x 2 cm, light brown, edematous and hemorrhagic, soft and smooth consistency. In the coronal section, the endocervical canal and uterine cavity are occupied by a placenta measuring 10 x 4 cm with cotyledons infiltrating the myometrium and in contact with the serosa. The umbilical cord with central insertion measures 3 x 1 cm and has three visible vascular structures.

Microscopic description

**Figure 6:**
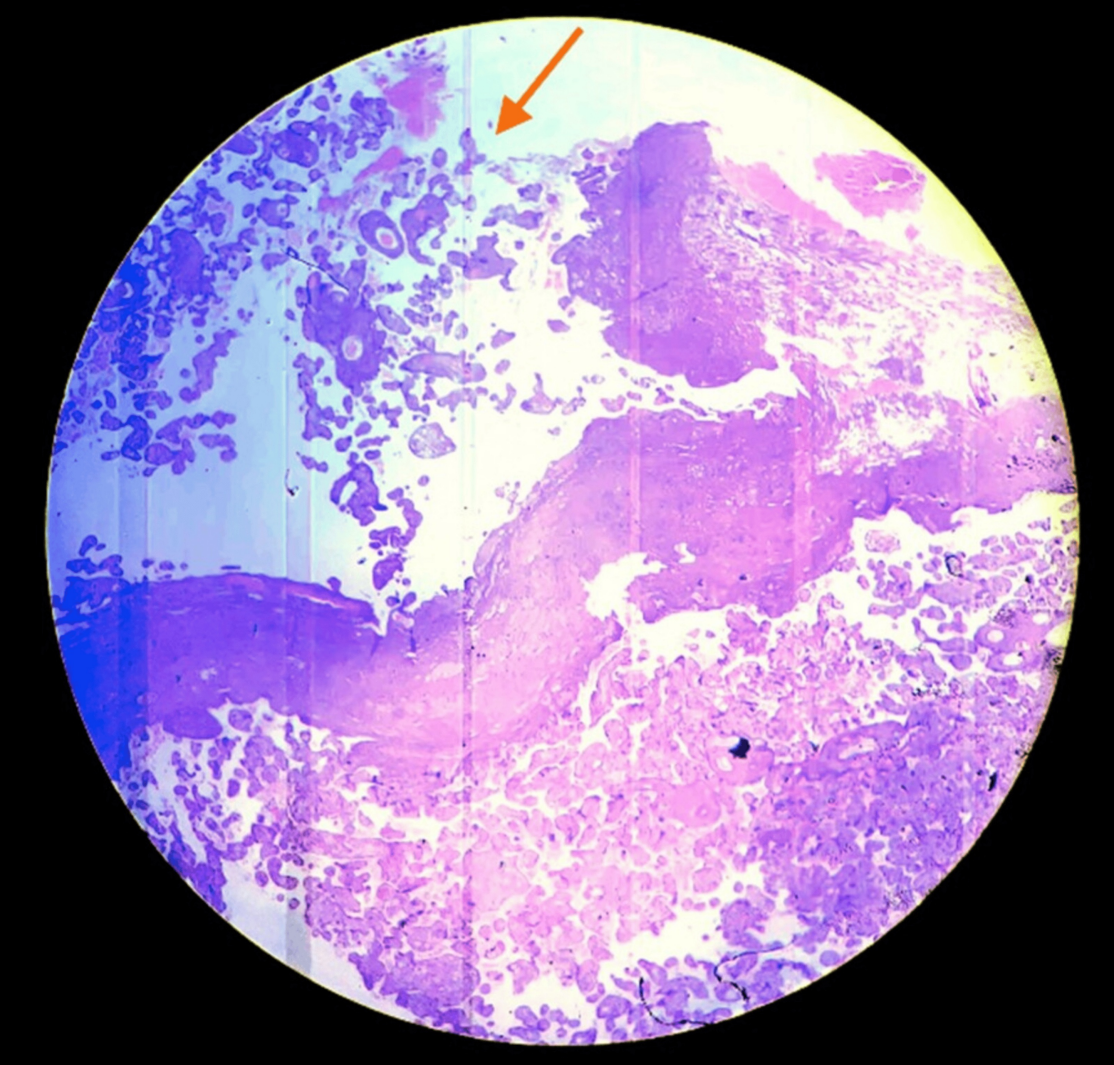
Histopathological examination (HE, 100x). Shows thinning and decidual loss, with placental villi in direct contact with the myometrium and outside of it, confirming the diagnosis of placenta percreta (arrow).

The histological image showed cervical tissue with the presence of a diffuse chronic inflammatory infiltrate. Villi of the placenta was seen infiltrating the entire myometrium and serosa with a final diagnosis of placenta percreta (Figure 6).

## Discussion

Placenta percreta is one of the most severe forms of abnormal adhesion of the placenta to the uterus and is part of a spectrum of disorders known as "placental accreta," which also includes placenta accreta and increta, accounting for 22% of these cases. It is characterized by the chorionic villi invading the entire thickness of the myometrium, perforating the serosa of the uterus, and extending to adjacent organs [4]. The most affected structures are the intestine and the bladder, the latter being involved in 1 in every 10,000 births [5].

Magnetic resonance imaging in patients with suspected placental accreta is a fundamental diagnostic tool that can dramatically influence the approach and treatment. It should ideally be performed between 28-32 weeks of gestation with a carefully designed protocol to obtain images in multiple planes (axial, coronal, and sagittal) to adequately assess the extent of placental invasion and obtain a three-dimensional view of the affected area [6].

Key considerations include T2-weighted images with and without fat saturation to delineate the thickness and continuity of the myometrium, as well as identify areas of placental invasion. In this sequence, the difference in tissue signal intensity (the placenta appearing hyperintense and the myometrium relatively more hypointense) allows for the identification of areas of invasion with loss of the retroplacental line [4].

T1-weighted sequences can detect intraplacental hemorrhages, which are common in placenta percreta. These sequences also help to visualize the interface between neighboring tissues and determine whether there is an extension of placental tissue into these tissues. Although the use of gadolinium is generally contraindicated during pregnancy due to concerns about fetal safety, in some severe cases it may be considered under specific circumstances. Gadolinium highlights blood vessels and may provide additional information about abnormal vascularization at the placental implantation site [7].

Diffusion-weighted imaging (DWI) is part of the protocol due to its usefulness in cases with myometrial congestion, where it is difficult to determine the limits of the placenta and the myometrium. Due to the intrinsic characteristics of each, the placental tissue behaves with high signal intensity, and the myometrium is of low intensity in sequences with high b values, which is useful to achieve an adequate differentiation between the two structures, even greater than that obtained in T2-weighted sequences [4].

Characteristic MRI findings in placental accreta, which are present in our case, include decreased thickness of the myometrium or its absence in areas where the placenta has invaded, disruption of the interface between the myometrium and the placenta, abnormal vascularization around the site of placental insertion, invasion into adjacent organs (bladder, as in our patient), and the presence of placental lacunae (hypointense areas on T2), which indicate dilated vascular spaces within the placenta [6].

Although bladder involvement is rare, its identification is important for the patient's outcome. A significant increase in massive bleeding during surgery has been described compared to cases where there is no involvement in this organ. The magnetic resonance study shows the interruption in the bladder wall in the T2 sequence and loss of the fat plane in the uterovesical region [8]. Due to the risks mentioned above, an early diagnosis by MRI and multidisciplinary management is essential, as was the case here, where it was decided to resolve the pregnancy via cesarean section and hysterectomy, presenting a favorable evolution after the intervention.

## Conclusions

MRI is especially relevant in the diagnosis of placental accreta when ultrasound is inconclusive or when placenta percreta is suspected. The ability of MRI to provide detailed, multiplanar images allows for accurate planning of obstetric management, significantly reducing associated risks such as massive hemorrhages and surgical complications.

The case presented shows the importance of imaging studies as part of multidisciplinary management coordination that led to adequate treatment of the pathology without major complications for the mother and the baby.
